# Factors affecting the utilisation of improved ventilated latrines among communities in Mtwara Rural District, Tanzania

**Published:** 2012-12-25

**Authors:** Koronel Kema, Innocent Semali, Serafina Mkuwa, Ignatio Kagonji, Florence Temu, Festus Ilako, Martin Mkuye

**Affiliations:** 1 African Medical and Research Foundation (AMREF), Tanzania; 2 Muhimbili University of Health and Allied Sciences (MUHAS), Tanzania

**Keywords:** Tanzania, utilization, Ventilated Improved Pit latrines, water and sanitation

## Abstract

**Introduction:**

The Tanzania government, working in partnership with other stakeholders implemented a community-based project aimed at increasing access to clean and safe water basic sanitation and promotion of personal hygiene in Mtwara Rural District. Mid-term evaluation revealed that progress had been made towards improved ventilated latrines; however, there was no adequate information on utilisation of these latrines and associated factors. This study was therefore conducted to establish the factors influencing the utilisation of these latrines.

**Methods:**

A cross-sectional study was conducted among 375 randomly selected households using a pre-tested questionnaire to determine whether the households owned improved ventilated latrines and how they utilised them.

**Resuls:**

About half (50.5%) of the households had an improved ventilated latrine and households with earnings of more than 50,000 Tanzanian Shillings were two times more likely to own an improved latrine than those that earned less (AOR 2.1, 95% CI=1.1-4.0, p= 0.034). The likelihood of owning an improved latrine was reduced by more than 60 percent for female-headed households (AOR=0.38; 95% CI=0.20-0.71; p=0.002). Furthermore, it was established that all members of a household were more likely to use a latrine if it was an improved ventilated latrine (AOR=2.4; 95% CI=1.1-5.1; p= 0.024).

**Conclusion:**

Findings suggest adoption of strategies to improve the wellbeing of households and deploying those who had acquired improved ventilated latrines as resource persons to help train others. Furthermore, efforts are needed to increase access to soft loans for disadvantaged members and increasing community participation.

## Introduction

The global burden of diseases is estimated at 1.4 billion disability-adjusted life years (DALY) lost annually, a parameter used in the disbursement of aid. Of this figure, a staggering 20 percent occur in Sub-Saharan Africa (SSA), which is home to less than 10 percent of the global population [1]. To respond to this disproportional DALY, Sub-Saharan African and other developing countries have adopted the eight Millennium Development Goals (MDGs) strategies. One of the MDGs focuses on halving the proportion of people without sustainable access to safe drinking water and basic sanitation [2, 3].

The declaration of the International Drinking Water Supply and Sanitation Decade launched in the1980s is an integral part of the global efforts seeking to enhance access to safe water and decent basic sanitation [4]. This declaration was aimed at increasing universal access to clean and safe drinking water and sanitation by 1990. This resolve prompted low-income countries in partnership with donors and international Non-Governmental Organization (NGOs) to implement its provision in accordance with their respective local national contexts. By the end of the decade, the proportion of people accessing clean water increased from 30 percent in 1980 to 63 percent in 1990 in the rural areas, and from 77 percent to 82 percent for urban centres [5]. However twenty years later, in 2010, the proportion of people with access to clean water declined to 47.9% in rural areas, and to 79.5% in urban areas [6].

Despite a notable increase in access to safe water, unsafe water and poor sanitation still account for a significant proportion of the burden of disease observed in low-income countries, accounting for 7.4 percent of the burden of disease among under-five children in South Africa and 2.7 percent globally [7]. Thus, the disease burden associated with poor water and sanitation in Sub-Saharan Africa is higher than the global average. In fact, several other studies have similarly reported that such burden of disease in low income countries accounted for 3.4 percent as compared to a far lower rate of 0.2 percent found in high income countries [8–10]. The socio-economic consequences of this burden of disease negatively impacted upon the children's school enrolment and their attendance, in addition to increasing poverty among the populace. Other effects include loss of income and increased demand on the already overwhelmed health systems of developing countries.

So far, interventions to address the impact of poor water and sanitation have been reported to have significantly reduced the impact, including the number of missed classes among enrolled school children [11]. In other words, interventions do exist to efficiently and effectively redress the situation, albeit with some qualification and varying success rates. Such interventions have the added advantage of limiting transmissions in endemic situations as well as in curtailing the spread of such diseases to other areas [12]. On the other hand, lack of meaningful and timely interventions tends to lead to the persistence of factors that facilitate and sustain the transmission of water-borne and sanitation-related diseases in families and communities [13]. Also, it is worth noting that the causes of the burden of disease associated with poor water and sanitation are not limited to diarrhoea but also intestinal parasitic infections, soil transmitted helminthic infections, ophthalmic infections, skin and respiratory infections [9, 13, 14].

Despite continued investments to increase access to safe water and improved sanitation, poor practices such as limited utilisation of sanitary facilities contaminates environments and water sources. A study conducted in Bangladesh reported that the presence of a family latrine does not necessarily significantly increase its use and, consequently, there was no reduction in the risk of infection among children [14]. This suggests that efforts to increase access to safe water and improved sanitation have to be coupled with strategies to promote appropriate utilisation of sanitary facilities. Furthermore, the availability and use of the toilet depends on maintenance practices of the latrines and cleanliness as well as the quality of housing and household compound [15].

In fact, the Millennium Development Goal 7 (MDG 7) was adopted to hasten access to safe water and basic sanitation and subsequent utilization [16]. The global commitment to achieving the MDGs was reinforced by the 58th session of the United Nations Assembly, which proclaimed 2005-2015 as the International Decade of Water [16]. This resolution implored countries’ sustained commitment towards achieving MDG 7 through community participation. In response to the global policy strategies, African governments adopted and ratified the 2002 Johannesburg Plan of Implementation and Agenda 21. These African regional policy strategies are aimed at cutting by half the proportion of people unable to access safe drinking water and basic sanitation.

In this regard, the Tanzania policy and implementation strategies included observing global sanitation promotion days at the local level. Tanzania has ratified both the global and regional policies, which were localised through appropriate national policy and planning. As part of its plan of action, Tanzania has implemented national campaigns in a bid to increase access to safe water and basic sanitation. These campaigns have included *Mtu ni Afya* (Man is Health), *Maji ni Uhai* (Water is Life) the national strategies to improve access to safe water and basic and improved sanitation attracted support from development partners through bilateral agreements and also the private sector. African Medical and Research Foundation (AMREF) in Tanzania is one of the stakeholders actively participating in national efforts aimed at improving access to safe water and basic sanitation.

AMREF has been working with the government in implementing a community-based project in Mtwara Rural District to improve access to improved latrines and other sanitation facilities since 2008. Project implementation involved interventions, monitoring and evaluation strategies starting with a baseline analysis undertaken in 2008. The mid-term project evaluation revealed that a high proportion (69.0%) of the households had acquired low cost or standard improved ventilated pit latrines from none at the time of the baseline study. Though it was less than the project target, it was still higher than the current (22.2%) national average for Tanzanian [6]. On the other hand, there was inadequate information on households’ utilisation pattern of these latrines. Consequently, this study was conducted to determine the effective utilisation of these latrines and associated factors.

## Methods

**Study setting:** This cross-sectional study was conducted in Mtwara Rural District, one of the six districts that constitute Mtwara Region located in the Southern part of Tanzania, bordering Malawi in the South and the Indian Ocean in the East. Mtwara Rural District has an area of 3,597 square kilometres sub-divided into 18 wards and 118 villages. Its population size was 203,480 with a population density of 53 people per square kilometre [17]. The AMREF Water and Sanitation Project was implemented in 40 villages. Six wards - Kiromba, Mnima, Njengwa, Nitekela, Mtiniko and Chawi - were randomly selected study.

This rural district has no district hospital, but has two health centres and 28 dispensaries, which provide both curative and preventive health services. Communicable disease such as diarrhoea, as well as the maternal, peri-natal and nutritional causes account for over 82 percent of the burden of disease[17]. The infant mortality estimated at 126 per 1,000 births and under-five mortality of 112 per 1,000 is much higher than the national average of 70 per 1,000 and 93 per 1,000 respectively (DHS 2010) due to the prevalence of these factors.

### Sample size and sampling

This study aimed at determining the proportion of households who had actually acquired improved pit-latrines. The figure was estimated to stand at 30 percent without improved latrine during mid-term evaluation. Using the formula for calculating sample size for cross-sectional studies, a sample size of 375 was obtained. Each study village contributed equally to the study sample size which was drawn randomly from the existing list of villages’ households list.

### Research assistants

Young people who had completed 12 years of schooling were recruited and trained as research assistants. The training of these research assistants was aimed at making them understand the purpose of the study, research processes, household entry procedure, questionnaire administration procedures and proper data management. They also participated in pre-testing the questionnaire to be used during training.

### Data collection

The research assistants were introduced to each of the households selected to take part in the study by the respective authorities of each village. Routinely, the household heads were informed on the purposes of the project, the benefits and the voluntary nature of participation. The heads of households were then requested to provide a written consent for their permission to participate in the study. The interview with the head of the household was then conducted in the privacy of the household premises. A pre-tested questionnaire was used to collect data on demographic and socio-economic characteristics, water sources as well as hygienic practices.

### Quality of latrines

Household respondents were asked to indicate whether the household owned a toilet and of what type. A visual inspection was then done to confirm the quality status of the latrine. During the inspection, the toilet was then categorised as Standard Ventilated Improved Pit latrine (SVIP), Low-cost Improved Ventilated Pit-Latrine (LVIP) and Traditional Pit-Latrine. The information was recorded on a standard form attached to the household questionnaire. This analysis combined the SVIPs and LVIPs to create a variable of improved latrines. Those outside these two types were considered “not improved”.

### Utilisation

The respondents were also asked to respond spontaneously to a question requiring them to state the household members who usually used toilets. The response options included father, mother, children, all members and none.

### Variables

The dependent variables of the study included ownership of an improved latrine categorized as ‘yes’ or ‘no’ and ‘all household members defecated in latrine’ categorised as ‘yes’ -coded as 1 and 0 if ‘not all members’. Independent variables included the age of the respondents, the sex of the head of household and family income sources.

### Ethical considerations

The protocol for this study was submitted to the National Institute of Medical Research (NIMR) Ethical Review Committee of Tanzania for approval before conducting the research. The final accepted protocol formed the basis of conducting the study. Each respondent was briefed on the aims of the study as required and subsequently signed a consent form prior to participating in the study.

### Data management and analysis

A supervisor reviewed all the forms completed each day, checked for completion, and other errors. Corrections were made the next day. The quantitative data generated were handled and entered into a computer using ACCESS database software. Data were checked for completeness before being cleaned and analysed using STATA software [18]. Frequency tables for the dependent and independent variables were generated before cross-tabulations were made. Chi-squared tests were used to determine the association between categorical variables and fisher's exact test was used where a cell had frequency less than 10. Bivariate and multivariate logistic regression analysis were then done to determine the crude Odds Ratio (OR) and adjusted Odds ratio (AOR) for the association of each selected independent variable with the utilisation of the latrine while controlling other variables. The significance level was defined as a p-value of less than 0.05.

## Results

There were 375 study participants. A high proportion (79.7%) of these respondents was male and more than half (54.8%) were aged 40-49, with only a few (28.7%) earning more than 50,000 Tanzanian Shillings a month (Table 1). Almost all the respondents (97.1%) had a latrine of one kind. However, only half (50.5%) had an improved latrine (Table 1). Table 2 presents the results of the bivariate analysis of the type of latrine and whether all household members used it. A high proportion (92.1%) of those with latrines reported that all household members used a latrine. It was also established that those who owned a VIP latrine fared much better; a significantly higher proportion (97.9%) of all household members used the latrine compared to those with unimproved types of latrines (89.9%).

**Table 1:** Household socio-demographic characteristics and ownership of latrines

Characteristics	Frequency	Percentage
**Sex**		
Men	299	79.7
Women	76	20.3
**Age group**		
<30	60	16.0
30-49	205	54.8
50 +	110	29.3
**Income <50000 Tsh**		
No	107	28.7
Yes	268	71.3
**Has latrine**		
No	10	2.6
Yes	365	97.1
**Type of latrine**		
VIP	47	12.8
Improved	143	38.9
Unimproved	178	48.4
**Source of income**		
Business	20	5.5
Farming	325	86.6
Employed	39	10.4
None	10	2.7

**Table 2:** Proportion of households where all members use latrine by type of latrine

Type of latrine	All household members use a latrine		Х^2^ and p-value
	No	Yes	
Has latrine	29(7.9)	335(92.1)	Х^2^ =50.5, p< 0.001
VIP	1 (2.1)	46 (97.9)	Х^2^= 4.949, p= 0.026*
Improved	12 (8.4)	131 (91.6)	Х^2^= 0.5460, p=0.460
Unimproved	18 (10.1)	160 (89.9)	Х^2^= 0.0282, p= 0.867

Fisher's exact test

The study also analysed the association between household characteristics and use of the latrine by all members of the household. Table 3 presents results of a bivariate analysis of such an association in relation to household characteristics. Households with a monthly income of less than 50,000Tanzanian Shillings had a lower proportion (54.5%) of all household members who used a latrine than those with a higher income (63.0%). The difference in this regard was statistically significant at p=0.002. Similarly, among female-headed households the use of the latrine by all household members was comparatively low at 35.5 percent compared to the performance of 54.2 percent of male-headed households. This difference was statistically significant at p=0.004. Neither did households headed by unemployed persons fare much better. In this category of households, a lower proportion (47.8%) of all household members used the latrine at their disposal than 74.4% among households (74.4%) whose heads were in gainful employment. Once more, the difference was statistically significant at p=0.002.

**Table 3:** Household characteristics and use of latrine by all household members

Characteristics	All household members use latrine		p-value
	No (%)	Yes (%)	
**Income less than 50,000 Tsh**			
No	40(37.0)	68(63.0)	Х^2^= 9.367
p= 0.002			
Yes	146(54.5)	122(45.5)	
**Sex of respondent**			
Male	137(45.8)	162(54.2)	Х^2^= 8.435
p= 0.004			
Female	49(64.5)	27(35.5)	
**Age group ( in years)**			
<30	30(50)	30(50)	Х^2^= 0.770
p=0.680			
30-49	98(45.4)	107(54.6)	
50 +	57(52.8)	53(48.2)	
**Source of income**			
Business			
No	172 (48.5)	182 (51.2)	Х^2^= 2.632
p=0.105			
Yes	14(66.7)	6 (30.0)	
**Farmers**			
No	21(42)	29 (58)	Х^2^= 1.287
p=0.257			
Yes	164(50.3)	161(49.4)	
**Employed**			
No	175(52.1)	161(47.8)	Х^2^= 9.883
p=0.002			
Yes	10(25.6)	29 (74.4)	
No income			
No	178 (48.6)	187 (51.1)	*Х^2^= 1.7556
p=0.185			
Yes	7(70.0)	3(30.0)	

* Fisher's exact test, Tsh: Tanzanian Shillings

### Logistic multivariate analysis

Multivariate logistic regression analysis was conducted to determine the factors significantly associated with household ownership of improved latrines. The factors that were found to positively and significantly influence ownership of improved latrine were monthly income of more than 50,000 Tanzanian Shillings. It was established that a monthly income of more than 50,000 Tanzanian Shillings doubled the likelihood of the household owning an improved latrine (AOR=2.1, 95% CI=1.05-4.01, p= 0.034). Moreover, awareness of the AMREF activities also increased the likelihood by almost nine times of the household owning an improved latrine (AOR=9.3; 95% CI= 4.39-20.1; p < 0-001). The full details of this have been presented in Table 4. From a gender perspective, it was established that the likelihood of owning an improved toilet was reduced by more than 60 percent for female-headed households (AOR=0.4; 95% CI=0.2-0.7; p < 0.002).

**Table 4:** Crude and adjusted OR (AOR) of ownership of improved latrine and its association with other factors

Household has improved latrine	OR	95% CI	p-value	AOR	95% CI	p-value
Age	1.0	0.9-1.1	0.390	0.98	0.97-1.0	0.125
Income more than 50,000 Tsh per month	2.0	1.1-3.5	0.017	2.06	1.06-4.0	0.034
Female respondent	0.5	0.3-0.8	0.004	0.38	0.21-0.7	0.002
Source of income farming	0.7	0.4-1.3	0.257	0.73	0.10-5.0	0.745
No source of income	0.4	0.1-2.1	0.288	0.09	0.01-1.5	0.095
Source of income employment	3.2	1.5-6.8	0.001	3.81	0.06-228.9	0.522
Source of income business	0.5	0.2-1.2	0.105	0.27	0.07-1.0	0.057
Aware of AMREF project interventions	9.1	4.3-18.9	0.000	9.28	4.31-19.9	<0.001

It is evident that there was increased ownership of improved latrines with time in the areas under study following the project intervention. However, the AMREF initiative also strived to promote latrines use by all members in the household. Thus, a multivariate analysis was done to determine the relationship between household factors and the use of latrine by all members of that household. The result shows that only two factors were significantly associated with increased use of latrine by all household members: an improved latrine (AOR=2.4; 95% CI=1.1-5.1; p=0.024) and higher age, which increased the likelihood of ownership by three percent (Table 5).

**Table 5:** Crude and adjusted OR (AOR) of use of toilet by all household members and its association with other variables

All household members use latrine	OR	95% CI	p-value	AOR	95% CI	p-value
Improved toilet	2.1	1.0-4.1	0.052	2.39	1.12-5.13	0.024
Age (years)	1.0	0.9-1-1.1	0.055	1.03	1.00-1.06	0.047
Income less than 50,000 shillings per month	0.7	0.3-1.7	0.355	1.49	0.40-5.52	0.552
Female-respondent	0.6	0.3-1.4	0.239	0.93	0.40-2.18	0.874
Source of income: Farming	0.8	0.2-2.3	0.640	0.41	0.03-5.10	0.487
Source of income: Formal Employment	0.7	0.3-1.9	0.511	0.17	0.01-29.38	0.498
Source income business	0.9	0.4-2.4	0.990	2.14	0.07-61.31	0.658
Aware of AMREF project interventions	2.0	0.7-5.7	0.220	2.1	0.6-7.6	0.248

## Discussion

The study found that at least half of the households (50.5%) owned an improved latrine and less than half (40.0%) of the households members used the facility. It was also established that households with monthly income of more than 50,000 Tanzanian Shillings and those aware of the AMREF project were more likely to own an improved latrine. Furthermore, it was established that improved latrine and the age of the head of household were factors found to be significantly associated with increased use of latrine by all household members.

The AMREF project implementation strategies aimed at increasing access to improved latrines included educational campaigns, provision of credit facilities, developing their villagers’ technical capacity and availing the required construction materials. Community members and leaders were deployed to educate and inform other members of the community on the available strategies and importance of their active participation in improving access to resources for constructing improved ventilated latrine. The gradual rise in the acquisition of improved ventilated latrines can be attributed to the time villagers needed to adjust to the new demands and initiate change in earnest. Moreover, there is the issue of mobilising resources for erecting an improved ventilated latrine, hence the gradual onset of improved latrine ownership in the community.

Suresh observed that there are five characteristics related to the adoption of a new idea, product or behaviour: 1) relative advantage of adopting the new behaviour; 2) the compatibility of the new behaviour; 3) complexity of adopting the behaviour; 4) observability of the adopted behaviour; and 5) triability, which offers an opportunity to experiment with the behaviour before adopting it on a sustainable scale [19]. The target population could also be slow in adopting the new behaviour because of their limited perception of its advantage, or any other reason that could limit triability. Since early adopters tend to be motivated, knowledgeable and believers, they could also contribute meaningfully to promoting strategies aimed at consolidating the acquisition of ventilated latrine, hence resulting in a multiplier effect that would make the project gains more widespread.

Although the AMREF community project in Mtwara Rural District strategies exposed all the members of the community to the project interventions, those who had a monthly income of more than 50,000 Tanzanian shillings and those in gainful employment had a higher likelihood of acquiring improved latrines than those with less income and those who were unemployed. This difference in terms of ownership of improved latrine by wellbeing could be explained in two ways. One, as the project interventions included acquisition of construction materials and, in some cases, involved provision of loans for purchasing materials and labour, those with better income had a better purchasing power and, hence, opted to purchase the materials or took loans since they had the capacity to pay back.

Two, those earning 50,000 or more could also be people with better access to information and had a better understanding of the project's benefits than the poorer families. As such, they could easily take on bold strategies associated with acquisition of improved latrines. In fact, some of them could have acquired improved ventilated latrines because they had a clear understanding and motivation to do so. Indeed, the study findings found that those who were classified as knowledgeable about the AMREF project also fared better in implementing its initiatives. These included the respondents who had responded to the call by the project early and took action as soon as they had contact with the project implementers.

Whereas the early project adopters were defined by their better income or wellbeing, the late converts were female-headed households. Generally, female-headed households experienced lower productivity and income due to the physical absence of a male partner input. Socially, patriarchal values tend to limit socio-economic opportunities in which women are involved in, including the acquisition of resources necessary to build improved latrines. In addition, females are more likely to have lower education since patriarchal values in many of these rural societies tend to favour the education of the male child. These odds of less income coupled with less education are evidently stacked against female-headed households and make it harder for them to acquire improved ventilated latrine than their male counterparts.

In addition to promoting access to improved latrines, the AMREF project in Mtwara Rural District also sought to promote its effective use to enhance the cumulative health benefits. In fact, it was established that members of a household were all more likely to use a latrine if their household had an improved ventilated latrine. The strong association between improved latrine and its use by all household members could be attributed to the conducive and hygienic environment a quality latrine offers to its users. Latrine improvement includes a latrine door, wall and roof which provide privacy even for adult males who would otherwise go to surrounding bushes to defecate and proper aeration.

## Conclusion

On the whole, the project registered reasonable gains in helping members of the community acquire ventilated improved latrines. The findings of this study suggest that the project's success is, however, undermined by selective uptake of the initiative by those with better income, hence the slow progress made with the poorer household, including female-headed households. Thus, the project's strategies should target those without or with little income, female-headed households and other vulnerable households. Some strategies in this regard could be increasing access to credit with easy terms that could be accessed by vulnerable households in addition to providing them with the necessary education. It is also possible to create a mechanism that would guarantee such female-headed households and other vulnerable poor households with access to credits. In addition to raising the ownership of improved latrines in the benefiting communities, the project also influenced change in personal hygiene and general sanitation behaviour. In the past, some members of the households defecated in bushes as the poor latrines in their homesteads lacked privacy. As a result, they were likely to spread water-borne diseases. Improved latrines have encouraged practically all household members to use these facilities when responding to both the short and long call of nature because of the issues of privacy, convenience and cleanliness. Thus, households should be encouraged and enabled to improve the quality of their latrines. However, without being economically empowered to do so, many of the poor households, including many female-headed households will continue finding it difficult to adopt the measures since their financial positions may not permit them to do otherwise. In other words, a mechanism that fosters the easy availability of soft credits coupled with more education provision and a concerted community that facilitates interaction and socio-economic networking can help to bring about the desired benefits even for the disadvantaged rural households.
